# Heat dissipation during hovering and forward flight in hummingbirds

**DOI:** 10.1098/rsos.150598

**Published:** 2015-12-16

**Authors:** Donald R. Powers, Bret W. Tobalske, J. Keaton Wilson, H. Arthur Woods, Keely R. Corder

**Affiliations:** 1 Department of Biology, George Fox University, Newberg, OR, USA; 2 Division of Biological Sciences, University of Montana, Missoula, MT, USA

**Keywords:** heat dissipation, heat balance, flight, plumage, hummingbirds

## Abstract

Flying animals generate large amounts of heat, which must be dissipated to avoid overheating. In birds, heat dissipation is complicated by feathers, which cover most body surfaces and retard heat loss. To understand how birds manage heat budgets during flight, it is critical to know how heat moves from the skin to the external environment. Hummingbirds are instructive because they fly at speeds from 0 to more than 12 m s^−1^, during which they transit from radiative to convective heat loss. We used infrared thermography and particle image velocimetry to test the effects of flight speed on heat loss from specific body regions in flying calliope hummingbirds (*Selasphorus calliope*). We measured heat flux in a carcass with and without plumage to test the effectiveness of the insulation layer. In flying hummingbirds, the highest thermal gradients occurred in key heat dissipation areas (HDAs) around the eyes, axial region and feet. Eye and axial surface temperatures were 8°C or more above air temperature, and remained relatively constant across speeds suggesting physiological regulation of skin surface temperature. During hovering, birds dangled their feet, which enhanced radiative heat loss. In addition, during hovering, near-body induced airflows from the wings were low except around the feet (approx. 2.5 m s^−1^), which probably enhanced convective heat loss. Axial HDA and maximum surface temperature exhibited a shallow U-shaped pattern across speeds, revealing a localized relationship with power production in flight in the HDA closest to the primary flight muscles. We conclude that hummingbirds actively alter routes of heat dissipation as a function of flight speed.

## 1. Introduction

Because flight muscles have low mechanical efficiency (10–15%), they produce large amounts of heat that must be lost in order for normal body temperature to be maintained [1,2]. Dissipating heat during flight is challenging for birds because the feathers that cover their body surface restrict heat loss during flight [3]. The few studies that have addressed heat loss during flight model it assuming the surface of the plumage (rather than the skin) to be the true outer surface of the bird and used infrared imaging cameras with lower resolution than what is currently available [4,5]. Calculation of total heat loss depends on precise measurements of surface area [4], which is difficult in birds, and on a good understanding of heat transfer across the plumage [3]. High-resolution infrared imaging can improve our understanding of the complex nature of heat transfer through the plumage, providing insight into how birds prevent overheating during flight.

Infrared (IR) thermography suggests that, during flight, most heat is lost from a few restricted areas. In European starlings (*Sturnus vulgaris*), surface heat loss was uneven and was the highest in key areas of heat loss during relatively fast flight from 6 to 14 m s^−1^ [4]. Key heat dissipation areas (HDAs) were the head, legs and feet, and the area around the shoulder joint, all of which averaged 5–8°C above ambient temperature (*T*_a_). Similar results were obtained for hovering Anna's hummingbirds (*Calypte anna*) with the addition that airflow induced by the wings increased heat loss from the torso [5]. An emphasis on heat loss through HDAs is consistent with the observation, in both studies, that the surface temperature (*T*_s_) of most plumage outside the HDAs was within 2°C of *T*_a_. Further, mean whole-body *T*_s_ was strongly correlated with *T*_a_, suggesting that most of the plumage surface gives off little metabolic heat and that a few focal areas (the HDAs) give off the vast majority of it.

Hummingbirds are unique in that they can fly at speeds ranging from 0 (hovering) to more than 12 m s^−1^. Such a large range may permit them to transition among different major routes of heat loss. During hovering, radiative and evaporative heat loss probably account for a higher proportion of heat dissipation than during faster flight, in which convective heat loss probably dominates. In this study, we used state-of-the-art IR thermography to examine surface heat loss in captive hummingbirds with the goal of understanding how they lose adequate heat over a broad range of flight speeds. We made the following predictions: (H1) HDAs will decrease in size with increasing flight speed as birds shift from radiative to convective heat transfer (H1a), but because power is known to vary according to a U-shaped curve, heat dissipation may also vary in the same manner with flight speed (H1b). (H2) Because HDAs occur in regions of low feather density, HDA *T*_s_ will be strongly influenced by skin *T*_s_. Because skin *T*_s_ is a function of local circulation [6] and metabolic heat conducted from the body core, HDA *T*_s_ will be a more accurate indicator for heat dissipation than mean body *T*_s_ which is heavily influenced by regions of high feather density.

## 2. Material and methods

### 2.1 Study species

Calliope hummingbirds (*Selasphorus calliope*; three males, 2.4–2.9 g; four females, 2.6–3.1 g) were collected during June 2010–2012 in Missoula, MT (Missoula County), USA. In the laboratory, birds were housed individually in 1×1×1*m* cages and fed a 50 : 50 mixture of 20% sucrose solution and Nectar Plus^©^ *ad libitum* using a Dr JB's hummingbird feeder. All study subjects maintained mass during captivity, confirming that the feeder solution adequately supplied essential dietary needs.

### 2.2 Infrared thermography

To measure *T*_s_ of *S. calliope* at flight speeds of 0–12 m s^−1^ with increments of 2 m s^−1^, we used an open-circuit, variable-speed wind tunnel, the properties of which have been described previously [7]. The working section of the tunnel is 85 cm in length, square in cross section, 60 × 60 cm at the inlet and increasing to 61.5 × 61.5 cm at the outlet to accommodate boundary-layer thickening. Maximum deviations in velocity within a cross section are less than 10% of the mean, the boundary layer is less than 1 cm thick and turbulence is 1.2%. Wind speed during experiments and for reporting in this paper is equivalent air velocity rather than true air velocity [8].

Infrared (IR) images were collected using a FLIR SC4000 or SC6700 IR video camera set to record at a frame rate of 300 Hz. Calibration images of a 12 cm ruler were recorded prior to each run to set the scale used for calculating HDA areas from single-frame images. Videos were recorded through a hole cut in the Plexiglas^r^ wall of the wind tunnel chamber. Hummingbirds were positioned for recording in the chamber using a 1.0 ml feeder made from a tuberculin syringe containing 20% sucrose solution. All recordings were a right-side, lateral view visualizing half the hummingbird's surface. We assumed emissivity was 0.95 across all surfaces of the hummingbirds [9]. For each recording, 6–10 single-frame images illustrating the end of both upstroke and downstroke were exported for analysis. Videos were recorded using ExaminIR (FLIR, Inc.) and single-frame images analysed using ImageJ (NIH).

Each single-frame image was analysed for mean and maximum surface temperature (*T*_s_, °C), and body area (mm^2^) that was more than 0.5°C ambient temperature (*T*_a_, °C). Selecting the minimum as *T*_a_+0.5°C eliminated noise effects of the boundary layer. Mean and maximum *T*_s_, and area were also measured for specific body regions that exhibited focused high *T*_s_ (eye, feet, shoulder and axial). For these specific regions, the minimum *T*_s_ threshold was 28°C as this was the minimum temperature at which the region boundaries were clearly defined.

### 2.3 Plumage insulation

We used an *S. calliope* carcass to measure the effect of feathers on insulation, adapting previously described methods [4]. We pinned a dead female hummingbird (obtained dead from the wild in an unrelated study) in a streamlined posture with the wings folded against the body, and we dried the specimen for 48 h using a Percival scientific drying oven (Model I-35LL, Percival, Inc., Boone, IA, USA) set at 47°C. Wings were dried folded against the body because of concern that the wings would break at high wind speeds if they were extended. Further, removal of the wings would create unrealistic airflow patterns. After the specimen was dry, we removed tissue from the coelom and skull using a scalpel, forceps and a drill. We then inserted a heating element into the cavities. The heating element was custom-made using high resistance nichrome wire (0.04 Ω cm^−1^; Omega Engineering, Inc., Stamford, CT, USA, NI60/CR16) encased in ovoids of PC-Marine epoxy putty (Protective Coating Co., Allentown, PA, USA). Three millimetre leads of nichrome wire extended laterally from the cranial and caudal regions of the carcass, and these were used to connect the specimen to an AC electronic circuit. We used a variac to supply power to the circuit (Powerstat Type 116, The Superior Electric Company, Bristol, CT, USA), and we measured amperage (I) and voltage (V) applied to this circuit using digital multimetres (I, Fluke 177, Fluke Corporation, Everett, WA, USA; V, RadioShack 22–163, RadioShack, Ft. Worth, TX, USA). The accuracy of the multimetres was verified with an Axon Instruments A/D board. We measured power (P) to maintain an average surface temperature on the specimen of 24°C (mean observed hummingbird plumage surface temperature) according to the formula: *P*=*VA*. The specimen was mounted to rigid insulated lead wires that were, in turn, supported by a wooden sling so that the specimen was oriented horizontally, parallel with incurrent flow, in the centre of the working section of the variable-speed wind tunnel. We conducted heating tests over a range of air velocities from 0 to 12 m s^−1^, first with the specimen intact, and then with all feathers removed. For tests on the carcass with the feathers removed average surface temperature was maintained at 36°C (maximum observed hummingbird skin temperature). To measure surface temperature, we used lateral-view thermal images. Ambient conditions during the tests were 22°C, 31% relative humidity and 906 hPa.

### 2.4 Particle image velocimetry

To measure airflow in the vicinity of the HDAs during hovering, we employed particle image velocimetry (PIV) using the techniques we have described in detail previously [10,11]. We sampled the near-field flow representing the middle of upstroke and the middle of downstroke. For mid-wing-induced velocity, we sampled frontal images of the wake, rooted at the shoulder, within two chord lengths of the wing, and our sample area was approximately 15×15 mm (approx. 1 body width). To sample air in the vicinity of the eye, shoulder and feet, we used approximately 5×5 mm sample areas within a parasagittal plane that intersected the shoulder. We measured air velocity immediately dorsal to the eye, dorsal to the shoulder and ventral to the feet. We report values for maximum air velocity (m s^−1^) within the sample areas, averaged among four birds.

### 2.5 Heat budget

Heat content (*H*) can be described by the following equation [12]:

2.1 $$H=M+Q_{a}-R-C-G-\lambda E,$$

where *M* is heat produced by metabolism, *Q*_a_ is radiation absorbed by the surface, *R* is infrared radiation emitted by the surface, *C* is heat gained or lost by convection, *G* is heat gained or lost by conduction and λ*E* is heat lost by evaporation. Changes in temperature can be estimated as changes in *H* divided by the heat capacity of the organism. For hummingbirds, we can simplify slightly by assuming that there is no heat exchange by conduction during flight. Thus, *G*=0 and

2.2 $$H=M+Q_{a}-R-C-\lambda E.$$

Details and assumptions used for calculation of *M*, *Q*_a_, *R* and λ*E* can be found in the electronic supplementary material.

We used three methods to estimate convection (*C*) in W.

**Figure 1.**
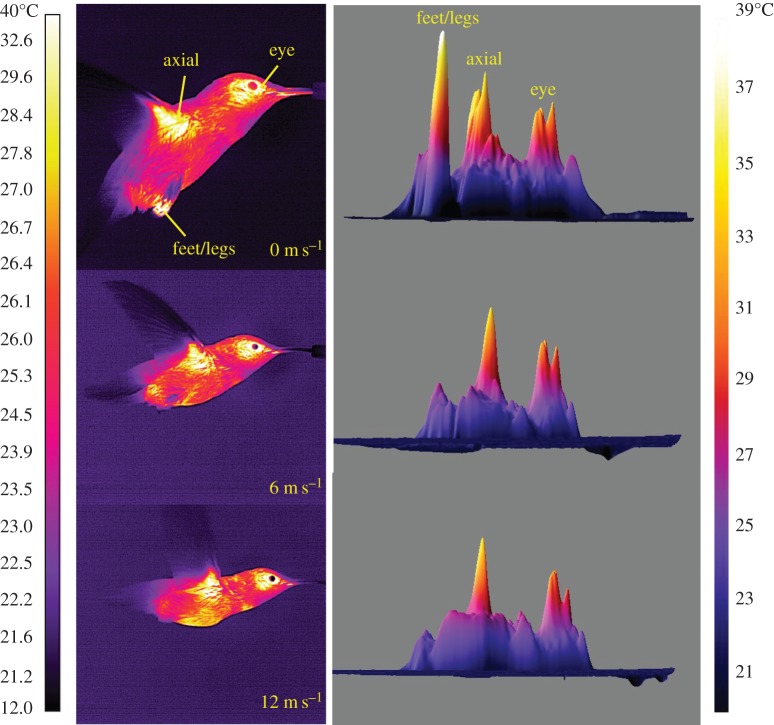
On the left are infrared (IR) thermographic images of *S. calliope* flying at 0 (hovering), 6 (intermediate speed) and 12 (high speed) m s^−1^. On the right are three-dimensional plots of surface temperature corresponding to each of the IR thermographic images. Eye and axial HDAs are visible across the full range of flight speeds. The feet/legs are extended and visible during hovering, but are most often retracted beneath the plumage during forward flight.

(i) We estimated convection directly using measurements of the power required to maintain the *T*_s_ of a calliope hummingbird carcass (*C*_*C*_) with full plumage at 24°C (*W*_p_; figure 1). Because the power required to maintain carcass *T*_s_ integrates both convective and radiative heat transfer, we calculated convective heat transfer as
  2.3 $$C_{C}=\;W_{p}-|\mathrm{Total}\;Q_{a}-\mathrm{Total}\;R|,$$
  resulting in *C*_*C*_ values of 0.28–1.48 W across all wind speeds.
(ii) We calculated convection using carcass *W*_p_ and *T*_s_ to measure the heat transfer coefficient (*h*) of a calliope hummingbird [4]. The value of *h* (W m^−2^ K^−1^) was calculated as
  2.4 $$h=\frac{\left(W_{p}-|\mathrm{Total}\;Q_{a}-\mathrm{Total}\;R|\right)}{A(T_{s}-T_{a})}.$$
  Convective heat loss (*C*_*h*_) was then calculated as
  2.5 $$C_{h}=hA(T_{s}-T_{a})$$
(iii) We calculated convection assuming a sphere (*C*_s_) equivalent in volume to a calliope hummingbird using standard methods previously described [12,13]. Estimations of convective heat loss using a spherical model have been shown to be within 20% of actual values for animals [13] although no bird species were tested.
   We first calculated the volume (*V*) of a hummingbird using the equation:
  2.6 $$V(m^{3})=\frac{m}{\rho },$$
  where *m* is mass (0.002.7 kg) and *ρ* is density (0.000784 kg m^−3^ [14]). We then calculated the characteristic dimension (*L*) assuming
  2.7 $$L(m)=V^{1/3}.$$
  Reynolds number (*Re*) was then calculated as
  2.8 $$Re=\frac{u\rho L}{\mu },$$
  where *u* is wind speed (m s^−1^), *ρ* is air density (1.07 kg m^−3^ in Missoula, MT, USA) and *μ* is dynamic viscosity (18.3×10^−6^ m^2^ s^−1^ for air at 20°C [12]). Next we calculated the Nusselt number (*Nu*) as
  2.9 $$Nu=0.34Re^{0.6}.$$
  We then calculated *h* for the sphere as
  2.10 $$h=Nu\left(\frac{k}{L}\right),$$
  where *k* is thermal conductivity (25.7×10^−3^ W m^−1°^ C^−1^ for air [12]). Lastly, *C*_S_ is calculated using equation (2.5).

### 2.6 Analysis

We used general linear mixed models (GLMM) to test for significant effects of wind speed upon *T*_s_ and the size of HDAs (GenStat, VSN International). Patterns of variation in *T*_s_ and HDA size were evaluated by examining back transformed means and standard errors from the GLMM model predictions.

## 3. Results

**Figure 2.**
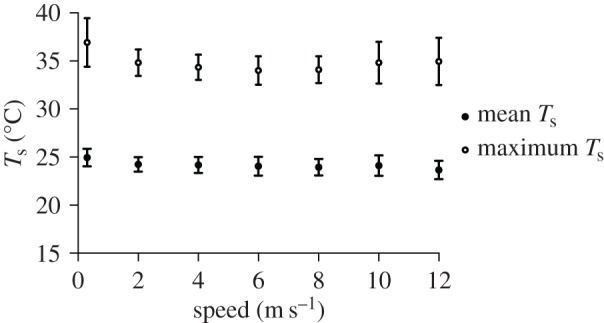
Average and maximum body *T*_s_ across flight speeds ranging from 0 to 12 m s^−1^. Values for 0 m s^−1^ are offset slightly for clarity. Average *T*_s_ declined slightly with flight speed while maximum *T*_s_ changed in a shallow U-shaped pattern.

Important HDAs in hummingbirds during flight included the area around the eye, the legs and feet, and the axial area under the wing (figure 1). Mean body *T*_s_ decreased significantly with speed (*F*_6,30_=5.42, *p*<0.001), most notably at 2 m s^−1^ (figure 2; electronic supplementary material, table S1). Total decrease in mean *T*_s_ between 0 and 12 m s^−1^ was only 1.3°C (range: 24.95–23.65°C). The differences between mean *T*_a_ (21.6±0.79°C) and mean *T*_s_ (*ΔT*) ranged from 3.4 to 2.1°C. Maximum *T*_s_ also changed significantly with speed and was highest at 0 and 12 m s^−1^ and lower at intermediate speeds (figure 2; *F*_6,35_=7.81,*p*<0.001; range 34.00–36.92°C).

**Figure 3.**
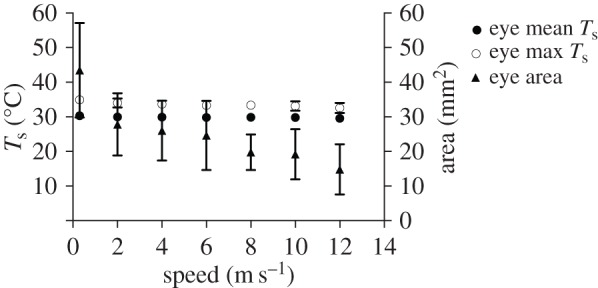
Eye HDA average and maximum *T*_s_, and area across flight speeds ranging from 0 to 12 m s^−1^. Average and maximum *T*_s_ decreased significantly between 0 and 2 m s^−1^, but remained constant above 2 m s^−1^. Area of the eye HDA decreased with flight speed.

The size of the eye HDA decreased significantly with speed (figure 3; electronic supplementary material, table S1; *F*_6,35_=32.43,*p*<0.001). The eye hot spot ranged from approximately 2 to 7% of the total visible body surface. Both mean eye *T*_s_ (*F*_6,28_=7.15,*p*<0.001; range 30.32–29.56°C; *ΔT*=8.0–8.7°C) and maximum eye *T*_s_ (*F*_6,35_=17.84,*p*<0.001; range 34.94–32.57°C) significantly decreased with speed with the model predicting the largest decrease in mean *T*_s_ (0.31°C) to occur between 2 and 4 m s^−1^ (figure 3; electronic supplementary material, table S1).

**Figure 4.**
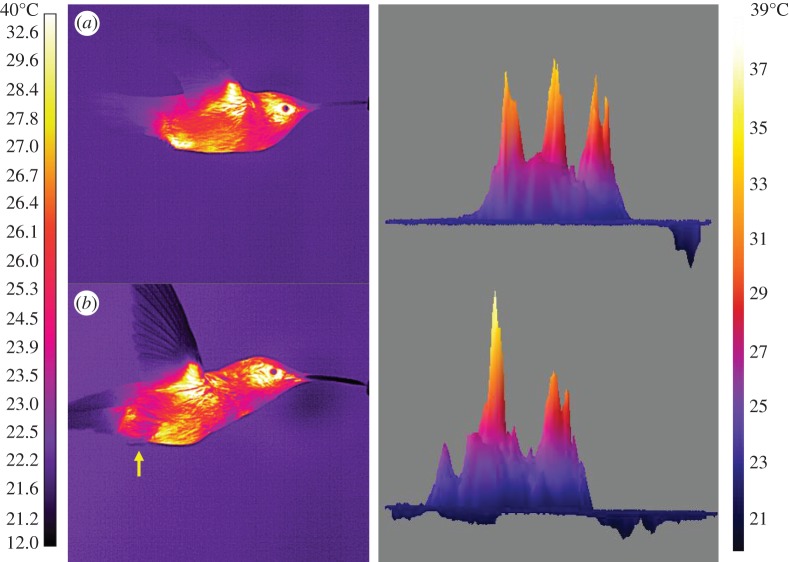
IR thermographic images and three-dimensional plots of surface temperature of *S. calliope* showing (*a*) high *T*_s_ region where feet are extended near, but not through the plumage at 10 m s^−1^ and (*b*) feet with *T*_s_ near *T*_a_ extended through the plumage at 12 m s^−1^.

Feet were consistently extended and visible in all birds at 0 m s^−1^ and in the minority of test subjects (2/7, 29%) at 2 m s^−1^. In two different birds at higher speeds (10–12 m s^−1^) feet were extended to near, but not through, the plumage surface (figure 4). An additional two birds extended their feet beyond the plumage surface, but unlike during hovering, the *T*_s_ of their feet and plumage surfaces were similar (figure 4). At 0 m s^−1^, the mean *T*_s_ of the feet was 30.19°C (*ΔT*=8.6°C), which was significantly higher than at other speeds where *T*_s_ was similar to the minimum *T*_s_ threshold (28°C; *F*_6,36_=4.33,*p*<0.001). Maximum foot *T*_s_ (33.47°C) was also significantly higher at 0 m s^−1^ than at other speeds (*F*_6,36_=14.06,*p*<0.001).

**Figure 5.**
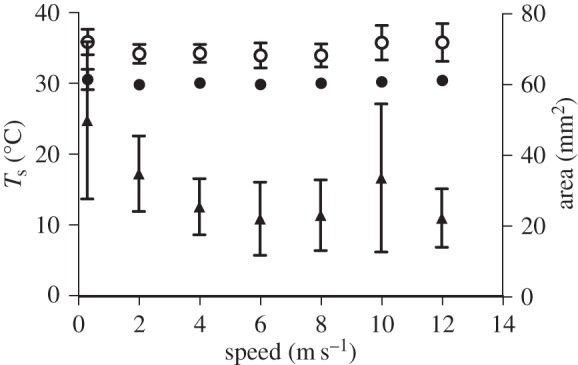
Axial HDA average (closed circles) and maximum (open circles) *T*_s_, and area (filled triangles) across flight speeds ranging from 0 to 12 m s^−1^. Average and maximum *T*_s_ differed significantly across wind speeds, but the differences were more pronounced in maximum *T*_s_, which varied in a U-shaped pattern. Area also differed significantly across wind speeds, also changing in a clear U-shaped pattern.

Mean *T*_s_ of the axial (under wing) HDA differed significantly with speed (*F*_6,30_=3.31,*p*=0.013) although the difference across speeds was subtle (figure 5; electronic supplementary material, table S1). Maximum axial *T*_s_ also differed across speeds (*F*_6,34_=4.47,*p*=0.002). Both mean and maximum *T*_s_ varied with wind speed in a ‘U’-shaped pattern similar to the mechanical and metabolic power curve for flight (electronic supplementary material, table S1) [15,16]. The area of the axial HDA differed with speed (*F*_6,34_=9.54,*p*=0.001) also in a U-shaped pattern except for a sharp increase at 10 m s^−1^ (figure 5; electronic supplementary material, table S1).

**Figure 6.**
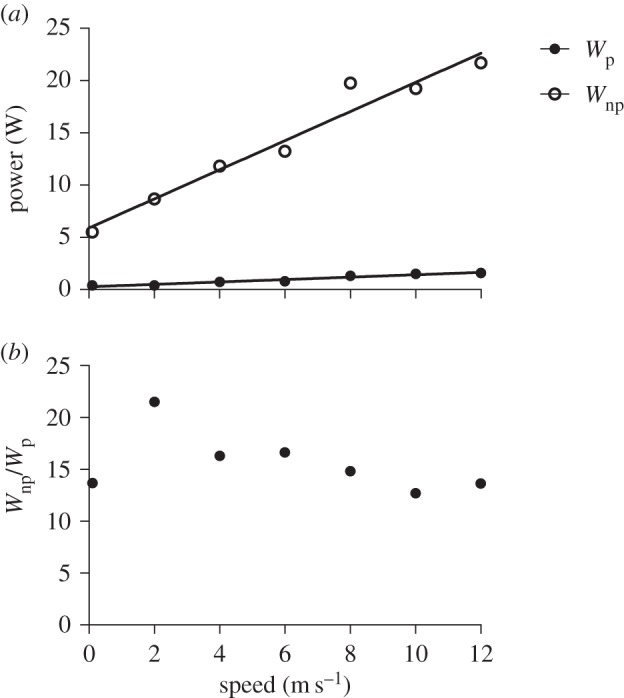
(*a*) Positive linear relationships of the power required to maintain an average surface temperature of 24°C (*W*_P_) in an *S. calliope* carcass with the plumage intact (*W*_P_=0.115*x*+0.274,*R*^2^=0.947, *F*_1,5_=88.96, *p*=0.0002), and an average skin temperature of 36°C (*W*_NP_) with the plumage removed (*W*_NP_= 1.394*x*+5.892,*R*^2^=0.956, *F*_1,5_=107.8, *p*=0.0001) over wind speeds ranging from 0 to 12 m s^−1^. (*b*) Ratio of *W*_NP_/*W*_P_ over wind speeds ranging from 0 to 12 m s^−1^.

Power required to maintain plumage *T*_s_=24°C in a feathered *S. calliope* carcass (*W*_p_) was positively related to wind speed (figure 6*a*) and ranged from 0.40 to 1.59 W. Much higher power was required in an *S. calliope* carcass with the plumage removed (*W*_np_, 5.50–21.70 W). The *W*_np_/*W*_p_ ratio was not correlated with wind speed, and varied from 12.7 to 16.6 for all speeds except 2 m s^−1^ at which the *W*_np_/*W*_p_ ratio was 21.5 (figure 6*b*). One possible explanation for this high ratio is that the heated carcass was positioned in the wind tunnel at an angle more appropriate for faster forward flight (greater than or equal to 4 m s^−1^) and did not accurately reproduce airflow patterns unique to 2 m s^−1^.

**Figure 7.**
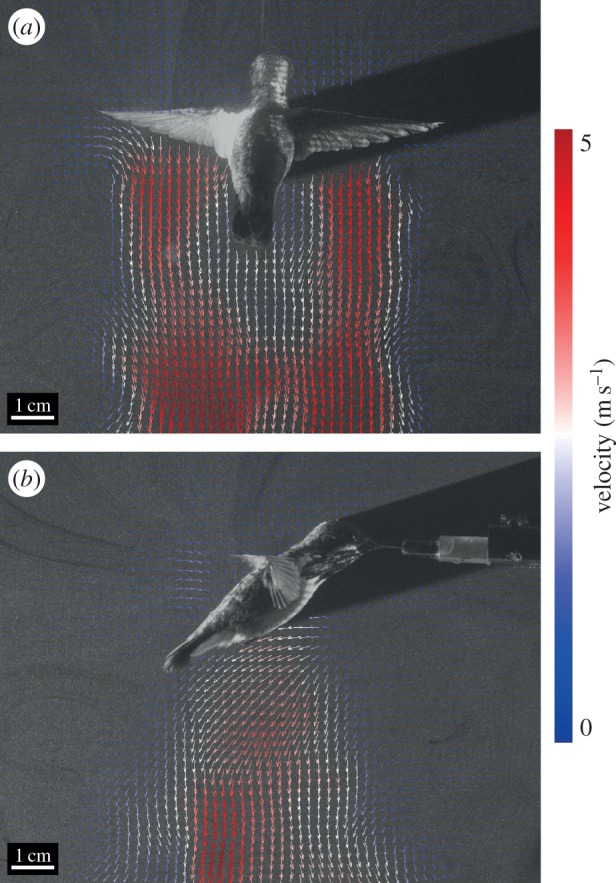
Dorsal (*a*) and right lateral (*b*) PIV images illustrating wake and near-field velocities at mid-wing for a male *S. calliope* in mid-upstroke of hovering flight.

Wake and near-field air velocities during hovering were greater during downstroke compared with upstroke (figure 7). At the mid-wing, peak induced velocities in the wake were 4.34±0.17 m s^−1^ for upstroke and 4.89±0.44 m s^−1^ for downstroke (figure 6*a*). Air velocity adjacent to the HDAs (figure 6*b*) was less than in the mid-wing wake and smallest at the eye (0.71±0.03 m s^−1^ and 0.73±0.13 m s^−1^ for upstroke and downstroke, respectively), intermediate at the shoulder (1.17±0.25 m s^−1^ and 1.45±0.19 m s^−1^) and largest at the feet (2.24±0.17 m s^−1^ and 2.83±0.26 m s^−1^).

Calculated heat budget parameters are found in table 1. Values for *H* varied substantially between models, with *H*_s_ most closely approximating heat balance. The large variation in *H* between models corresponded to differences in calculated values of *C* between methods, which could be as much as 5× at higher flight speeds.

**Table 1.** Calculated heat budget parameters across all wind speeds measured in this study. Units for all values is W unless otherwise specified.

speed (m s^−1^)	*M*	Total *Q*_a_	Total *R*	*C*_C_	*C*_S_	*C*_h_	λ*E*	*H*_C_	*H*_S_	*H*_h_
0	0.63	0.56	0.68	0.28	0.15	0.20	0.19	−0.03	0.10	0.06
2	0.35	0.56	0.67	0.29	0.14	0.21	0.19	−0.28	−0.13	−0.20
4	0.33	0.56	0.67	0.61	0.21	0.47	0.19	−0.62	−0.22	−0.47
6	0.34	0.56	0.67	0.68	0.26	0.52	0.19	−0.68	−0.25	−0.52
8	0.31	0.56	0.67	1.22	0.29	0.95	0.19	−1.24	−0.31	−0.98
10	0.37	0.56	0.67	1.40	0.36	1.10	0.19	−1.37	−0.33	−1.07
12	0.47	0.56	0.67	1.48	0.33	1.16	0.19	−1.36	−0.20	−1.04

## 4. Discussion

Our data suggest hummingbirds dissipate large portions of heat generated in flight through specific HDAs on their surface. Because hummingbirds are covered by plumage evolved to restrict heat loss in other contexts, they might have difficulty ridding themselves of the excess heat produced during flight, particularly at high ambient temperatures and while hovering [5]. During flight in *S. calliope*, most heat is lost through specific regions around the eyes, shoulder joint and feet. Because of shifts in the relative importance of radiative heat transfer and convection across flight speeds, maintaining heat balance will require physiological adjustments in regions where heat loss is high. The high-resolution IR imaging used in this study improves our understanding of how heat loss is restricted during flight to specific regions and the dynamic nature of these regions.

The use of the feet only at slow flight speeds (0–2 m s^−1^), and reduction in size of the eye and axial HDAs with increasing flight speed, supports our prediction that heat dissipated by hummingbirds during flight shifts from radiative and convective (hovering only) at slower flight speeds to primarily convective at faster flight speeds (H1a). Further, the shallow U-shaped pattern in maximum *T*_s_ and area in the axial HDA matches the U-shaped pattern observed for mechanical and metabolic power output during flight (electronic supplementary material, figure S1; H1b). However, because this pattern appears confined to the axial HDA, the link between heat loss and power output could be a localized effect due to proximity of this HDA to the primary flight muscles (pectoralis and supracoracoideus). Finally, relatively high, stable mean HDA *T*_s_ across flight speeds compared with the more densely plumaged and insulated general body surfaces is consistent with our prediction that HDA *T*_s_ is largely a reflection of skin *T*_s_ and that HDAs provide a better thermal gradient for heat transfer than the more densely plumaged general body surfaces (H2). Decreasing size of HDAs with flight speed could in part result from localized cutaneous regulation of circulation to match heat delivered to the HDA surfaces with the level of convective heat transfer [6], but that was not tested in the current study.

**Figure 8.**
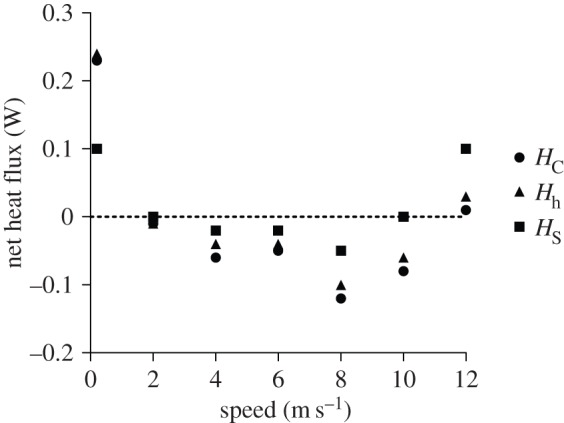
Estimates of net heat flux for *S. calliope*across tested wind speeds assuming heat transfer occurs only across HDAs (approx. 8% of total surface area). Circles are values for *H*_C_ (method 1), triangles are values for *H*_h_ (method 2) and squares are values for *H*_S_ (method 3). The dotted line represents the condition of perfect heat balance.

Metabolic power required for flight by *S. calliope* ranges from 0.33 to 0.63 W depending on flight speed, with the highest power required for hovering (electronic supplementary material, figure S1). The mechanical efficiency of flight muscles in hummingbirds is about 10% [1,5], suggesting that 0.30–0.57 W is converted to metabolic heat that must be lost during flight. At 0 m s^−1^, our models reasonably predicted net heat balance in that net heat flux never deviates more than 0.25 W from zero. However, as flight speed increases, net heat flux became increasingly more negative (in one case *H*=−1.36 W at 12 m s^−1^) largely due to the unrealistic estimates of convective heat loss (table 1) that resulted from the assumption of uniform heat transfer across all body surfaces. This is probably a poor assumption since our data suggest that most heat is lost during flight through specific HDAs. We therefore reran the models assuming heat transfer occurs only through HDAs (roughly 8% of the body surfaces), which resulted in net heat flux being close to 0 (predicting heat balance) for all forward flight speeds (figure 8).

For *S. calliope*, we estimate that insulation by plumage can reduce the rate of metabolic heat loss 13–17× across all flight speeds measured (figure 6*a*). Because the plumage is such a good insulator, most heat lost during flight occurs across a few HDAs where feather density is low or feathers are absent. The major HDAs observed in this study are similar to those observed in Anna's hummingbird (*C. anna*) during hovering [5], and in European starlings (*Sturnus vulgaris*) during forward flight [4]. Using sparsely feathered HDAs for heat loss makes sense, because birds probably have some physiological control over vascular heat delivery to these surfaces. In flying birds, the relationship between cutaneous blood flow and heat dissipation has received little study. By contrast, in laying hens, blood flow to unfeathered regions of skin increased 20× when birds were exposed to warm temperatures compared to only 5× in feathered regions [17]. Localized control of cutaneous heat loss has been observed in pigeons, which can elevate capillary blood flow under hormonal control [18]. More comparative work is needed to determine whether other avian species use similar mechanisms. There is some evidence from human studies that autoregulatory mechanisms control localized cutaneous blood flow [6]. If such mechanisms exist in birds it could enable rapid response to changes in the thermal gradient and convective conditions.

The HDA around the eye appears particularly important for hummingbirds. The width of the head is nearly maximal at the eyes, and this shape probably induces high near-field flow velocities around the eyes [19]. This region could therefore play a major role in eliminating excess heat produced during flight. The size of the eye HDA decreases 3× as hummingbirds transition from higher radiative heat loss during hovering and slow flight speeds to increased convection during faster flight (figure 3). During hovering, the thermal gradient between the general body plumage *T*_s_ and *T*_a_ (21°C) was less than 5°C, but between eye HDA *T*_s_ and *T*_a_ was approximately 15°C, which is consistent with the size of these gradients in pigeons [20]. The large gradient between the eye HDA *T*_s_ and the environment will result in a higher rate of heat flux. The decrease in size of the eye HDA is probably due to more rapid convective heat loss at higher flight speeds, but might also be influenced by regulation of blood circulation at the skin surface. The relatively high *ΔT* of the axial region was exceeded only by eye HDA, emphasizing that the axial area is also important for heat loss during flight. This is consistent with measurements made on *S. vulgaris* (*ΔT*=6°C at *T*_a_=21°C) [4]. Heat loss from this region is strongly influenced by muscle activity specifically associated with flight (i.e. U-shaped pattern, figure 5), as it overlies the massive pectoralis and supracoracoideus muscles, the primary muscles that power flapping flight.

During hovering flight, *S. calliope* dangled their feet to enhance heat loss (figure 4) since the lack of forward movement reduces convective heat losses from other exposed areas. At higher speeds, the feet were retracted below the plumage, presumably to reduce drag. *Calypte anna* dangled their legs and feet more frequently when *T*_a_ was higher, suggesting that dangling is a form of behavioural thermoregulation [21]. Large birds use a different strategy in that they are compelled to extend their legs parallel to the body to minimize drag and/or maintain stability because they are too large to retract [22,23]. In *S. vulgaris*, the legs are dangled at higher flight speeds (e.g. 10 m s^−1^), and individuals trade-off higher drag for the sake of increased heat loss [4]. In hummingbirds flying fast, the feet and legs typically remain retracted below the plumage, but in a few cases the external plumage surface below the legs is notably warmer than surrounding areas (figure 4). It is possible that the feet are in close contact with the inner surface of the plumage promoting more rapid conductive heat transfer to the exterior surface. This might be a means of further increasing convective heat loss without compromising aerodynamic form.

Hovering flight induces air flow along the torso of a hummingbird's body [10]. In *C. anna* induced airflow velocity was estimated to be 4.5 m s^−1^ [5]. While this is similar to the airflow velocity measured for *S. calliope* at mid-wing (figure 7), airflow near the surfaces of key *S. calliope* HDAs was substantially less. Thus, the degree to which this induced airflow interacts with HDAs is less clear. Measurements of airflow around the feet resulting from induced air flow could approach 3 m s^−1^, which could promote heat loss and compensate for the lack of convective heat loss over the rest of the body that otherwise occurs at faster flight speeds.

## Supplementary Material

Heat Budget Description

## Supplementary Material

Table S1 Back Transformed Means

## Supplementary Material

Table S2 Convection from Sphere Calculations

## Supplementary Material

Figure S1 Power Curve for Flight

## Data accessibility

All data are available in the electronic supplementary material and in Dryad: http://dx.doi.org/10.5061/dryad.8ck28.
