# Supplementary material for: Heat dissipation during hovering and forward flight in hummingbirds
Source: R Soc Open Sci. 2015 Dec 16;2(12):150598. doi: 10.1098/rsos.150598 (PMC4807464; doi:10.1098/rsos.150598)
Supplement: Heat Budget Description [file rsos150598supp1.pdf]

Estimated Heat Budget for the Calliope Hummingbird (*Selasphorus calliope*)

Methods and Calculations

An organism's heat content (H) can be described by the following equation [1]:

$$H = M + Q_a - R - C - G - \lambda E \qquad \text{(Eq. 1)}$$

where M is heat produced by metabolism, Q_a is radiation absorbed by the surface, R is infrared radiation emitted by the surface, C is heat gained or lost by convection, G is heat gained or lost by conduction, and λE is heat lost by evaporation. Changes in temperature can be estimated as changes in H divided by the heat capacity of the organism.

For hummingbirds, we can simplify slightly by assuming that there is no heat exchange by conduction during flight. Thus G = 0 and:

$$H = M + Q_a - R - C - \lambda E \qquad \text{(Eq. 2)}$$

Metabolic rate during flight (Q_{MR}) was measured directly using open-flow respirometry and exhibited a U-shaped pattern (figure S1) consistent with measurements made on other hummingbird species [2]. Metabolic heat production is calculated as:

$$M\ (\mathrm{W}) = Q_{MR} - Q_{ME} \qquad \text{(Eq. 3)}$$

where Q_{ME} is the mechanical work output for flight. We assume $Q_{ME} = Q_{MR}*0.1$ based on previous measurements of the mechanical efficiency of hummingbird flight muscles [3, 4].

Q_a and R ($\mathrm{W/m^2}$) were estimated using the Stefan Boltzmann law:

$$R\ (or\ Qa) = \varepsilon \sigma T^4 \qquad \text{(Eq. 4)}$$

where ε is emissivity, σ is the Boltzmann constant (5.673×10^{-8} W m^{-2} K^{-4}), and T is temperature in Kelvins.

For calculation of Q_a if we assume that all incoming radiation is from the Plexiglas © chamber surround the hummingbirds, and that the temperature of the Plexiglas is equal to ambient temperature (T_a = 21.6 °C or 294.75 K), and emissivity of Plexiglas (ε_P = 0.86; http://www.thermoworks.com/emissivity_table.html), then Q_a = 368.24 W/m^2. We calculate total incoming radiation as:

$$Total\ Q_a\ (W) = Q_a\ \varepsilon_H\ A \quad \text{(Eq. 5)}$$

where ε_H is the emissivity of hummingbird surfaces, and A is the total surface area of a calliope hummingbird. If we assume ε_H = 0.95 for both feather and skin surfaces [5, 6], and A = 0.0016 m^2 based on an allometric estimate of plumage surface area [7], then *Total* Q_a = 0.56W.

For calculation of R if we use mean body surface temperature (T_s), which varied from 23.65-24.95 °C (296.8-298.1 K) depending on wind speed, then R ranges from 418.21-425.56 W/m^2. We calculate total emitted radiation as:

$$Total\ R\ (W) = RA \quad \text{(Eq. 6)}$$

resulting in *Total R* values of 0.67-0.68W across all wind speeds.

For calculation of λE we estimated heat dissipated by respiratory evaporative water loss (REWL) during based on measurements of free-living hovering black-chinned hummingbirds (*Archilochus alexandri*, ~3 g) at 21 °C (0.095W) [8]. We then assumed that assumed that accounted for 50% of total evaporative water loss (EWL) [9] so that total heat dissipated by EWL was 0.19W. It is likely that REWL is lower during forward flight so this value could result in a small over estimation of H.

We used three methods to estimate convection (C) in W.

1) We estimate convection directly using measurements of the power required to maintain the T_s of a calliope hummingbird carcass (C_C) with full plumage at 24 °C (*Wp*; see figure 1). Since the power required to maintain carcass T_s integrates both convective and radiative heat transfer we calculated convective heat transfer as:

$$C_C = Wp - | Total\ Q_a - Total\ R | \qquad \text{(Eq. 7)}$$

resulting in C_C values of 0.28-1.48W across all wind speeds.

2) We calculated convection using carcass W_p and T_s to measure the heat transfer coefficient (h) of a calliope hummingbird [10]. The value of h ($Wm^{-2}K^{-1}$) was calculated as:

$$h = (Wp - | Total\ Q_a - Total\ R |) / A(T_s - T_a) \qquad \text{(Eq. 8)}$$

Convective heat loss (C_h) was then calculated as:

$$C_h = hA(T_s - T_a) \qquad \text{(Eq. 9)}$$

3) We calculated convection assuming a sphere (C_S) equivalent in volume to a calliope hummingbird using standard methods previously described [1, 11]. Estimations of convective heat loss using a spherical model have been shown to be within 20% of actual values for animals [11] although no bird species were tested.

We first calculated the volume (V) of a hummingbird using the equation:

$$V\ (m^3) = m/\rho \qquad \text{(Eq. 10)}$$

where m is mass (2.7 g) and ρ is density (0.784 g/cm^3 [12]). We then calculated the characteristic dimension (L) assuming:

$$L\ (m) = V^{1/3} \qquad \text{(Eq. 11)}$$

Reynolds number (Re) was then calculated as:

$$Re = = u\rho L / \mu \qquad \text{(Eq. 12)}$$

where u is wind speed (m/s), ρ is air density (1.07 kg m^{-3} in Missoula, MT, USA) and μ is dynamic viscosity (18.3 x 10^{-6} m^2/s for air at 20 ^{0}C [1]). Next we calculated the Nusselt number (Nu) as:

$Nu = 0.34Re^{0.6}$ (Eq. 13)

We then calculated h for the sphere as:

$h = Nu\ (k\ /\ L)$ (Eq. 14)

where k is thermal conductivity (25.7 x 10^{-3} Wm^{-1}°C^{-1} for air [1]). Lastly, C_S is calculated using Eq. 9.

Summary

While our heat-budget models reasonably predicted heat balance (H = 0) at 0 m/s (hovering), when methods 1 and 2 were used to estimate C, H became progressively more negative during forward flight as flight speed increased (table S1). The extreme negative values for H are unrealistic in that they would result in rates of heat loss that the hummingbirds could not survive. These results illustrate the complex nature of heat transfer across the plumage surfaces of hummingbirds, and likely birds in general. When we modeled H assuming a sphere of equal volume to the hummingbirds predictions for forward flight were much closer to net heat balance (tables S1, S2). Finally, we reran the models assuming heat transfer during flight occurs only across HDAs (~8% of total surface area) resulting in all models predicting heat balance during forward flight. Interestingly, in this case highest deviation from heat balance occurs at 0 m/s suggesting that all convective heat transfer during hovering has not been quantified.

Open-flow respirometry methods

Hummingbird hovering and flight metabolic rate was measured in a wind tunnel at wind speeds ranging from 0-12 m/s (2 m/s intervals) using open-flow respirometry. See METHODS for a detailed description of the wind tunnel. We used a negative-pressure open-flow system modified as described below from that previously described in the literature [13, 14]. Expired respiratory gas was collected from hummingbirds feeding during hovering/forward flight inside a mask attached to a feeder constructed from a 1.0 mL luer-tip syringe. The expired gas was pulled through a humidity sensor (RH-100; Sable Systems, Las Vegas, NV), CO_2 analyzer (Foxbox; Sable Systems), and O_2 analyzer (Oxzilla; Sable Systems) by a mass-flow system (MFS-2; Sable Systems) along with ambient air at a flow rate of 3 L/min. Output from the humidity sensor and CO_2 analyzer were used to mathematically calculate dry, CO_2-free flow rate [15]. Data acquisition and analysis was done using Warthog LabHelper and LabAnalyst software (Mark Chappell, UC Riverside) running on an Apple MacBook Pro. Gas measurements were used to calculate O_2 consumption (VO_2) rate (mL/min). Because feeding bouts were typically too short to achieve steady-state VO_2, total O_2 consumed (mL) was determined by integration of the VO_2 curve, then dividing by the total feeding time [13]. Feeding time was calculated from video recordings (Redlake PCI-500; Redlake MASD LLC, San Diego, CA, USA) of feeding bouts. VO_2 can be used to estimate the energetic cost of hovering/forward flight (kJ). The energy equivalent of VO_2 will be calculated using the standard conversion of 1L O_2 = 20.1 kJ.

Literature Cited

[1] Gates, D.M. 1980 *Biophysical ecology / David M. Gates*. New York, Springer-Verlag.

[2] Clark, C.J. & Dudley, R. 2010 Hovering and Forward Flight Energetics in Anna's and Allen's Hummingbirds. *Physiological and Biochemical Zoology* **83**, 654-662. (doi:doi:10.1086/653477).

[3] Wells, D.J. 1993 Muscle Performance in Hovering Hummingbirds. *Journal of Experimental Biology* **178**, 39-57.

[4] Evangelista, D., Fernandez, M.-J., Berns, M.S., Hoover, A. & Dudley, R. 2010 Hovering Energetics and Thermal Balance in Anna's Hummingbirds (Calypte anna). *Physiological and Biochemical Zoology* **83**, 406-413. (doi:doi:10.1086/651460).

[5] Birkebak, R.C., Birkebak, R.C. & Warner, D.W. 1964 A Note on Total Emittance of Animal Integuments. *Journal of Heat Transfer* **86**, 287-288. (doi:10.1115/1.3687122).

[6] Cossins, A.R. & Bowler, K. 1987 *Temperature biology of animals*. London; New York, Chapman and Hall.

[7] WALSBERG, G.E. 1978 The Relationship of the External Surface Area of Birds to Skin Surface Area and Body Mass. *Journal of Experimental Biology* **76**, 185-189.

[8] Powers, D.R., Getsinger, P.W., Tobalske, B.W., Wethington, S.M., Powers, S.D. & Warrick, D.R. 2012 Respiratory evaporative water loss during hovering and forward flight in hummingbirds. *Comparative Biochemistry and Physiology a-Molecular & Integrative Physiology* **161**, 279-285. (doi:10.1016/j.cbpa.2011.11.008).

[9] Bernstein, M.H. 1971 Cutaneous Water Loss in Small Birds. *The Condor* **73**, 468-469.

[10] Ward, S., Rayner, J.M.V., Möller, U., Jackson, D.M., Nachtigall, W. & Speakman, J.R. 1999 Heat transfer from starlings sturnus vulgaris during flight. *The Journal of Experimental Biology* **202**, 1589-1602.

[11] Mitchell, J.W. 1976 Heat transfer from spheres and other animal forms. **16**, 561-569.

[12] Dubach, M. 1981 Quantitative analysis of the respiratory system of the house sparrow, budgerigar and violet-eared hummingbird. *Respiration Physiology* **46**, 43-60.

[13] Bartholomew, G.A. & Lighton, J.R. 1986 Oxygen consumption during hover-feeding in free-ranging Anna hummingbirds. *Journal of Experimental Biology* **123**, 191-199.

[14] Welch Jr, K.C. 2011 The power of feeder-mask respirometry as a method for examining hummingbird energetics. *Comparative Biochemistry and Physiology Part A: Molecular & Integrative Physiology* **158**, 276-286. (doi:http://dx.doi.org/10.1016/j.cbpa.2010.07.014).

[15] Lighton, J.R.B. 2008 *Measuring Metabolic Rates : A Manual for Scientists: A Manual for Scientists*, Oxford University Press, USA.

Table S1. Calculated heat budget parameters across all wind measured in this study. Units for all values is W unless otherwise specified.

Speed (m/s)	M	*Total* Q_a	*Total R*	C_C	C_S	C_h	λE		H_C	H_S	H_h
0	0.63	0.56	0.68	0.28	0.00	0.20	0.19		-0.03	0.26	0.06
2	0.35	0.56	0.67	0.29	0.15	0.21	0.19		-0.28	-0.22	-0.20
4	0.33	0.56	0.67	0.61	0.23	0.47	0.19		-0.62	-0.35	-0.47
6	0.34	0.56	0.67	0.68	0.27	0.52	0.19		-0.68	-0.41	-0.52
8	0.31	0.56	0.67	1.22	0.31	0.95	0.19		-1.24	-0.50	-0.98
10	0.37	0.56	0.67	1.40	0.38	1.10	0.19		-1.37	-0.55	-1.07
12	0.47	0.56	0.67	1.48	0.35	1.16	0.19		-1.36	-0.40	-1.04

Table S2. Parameter estimations for calculation of C_S.

v (m/s)	Re	Nu	h ($Wm^{-2}K^{-1}$)	T_s (°C)
0	0	0.00	0.00	24.95
2	1766	32.26	54.91	24.24
4	3532	48.90	83.22	24.17
6	5297	62.37	106.14	24.05
8	7063	74.11	126.14	23.94
10	8829	84.73	144.21	24.11
12	10595	94.53	160.88	23.65

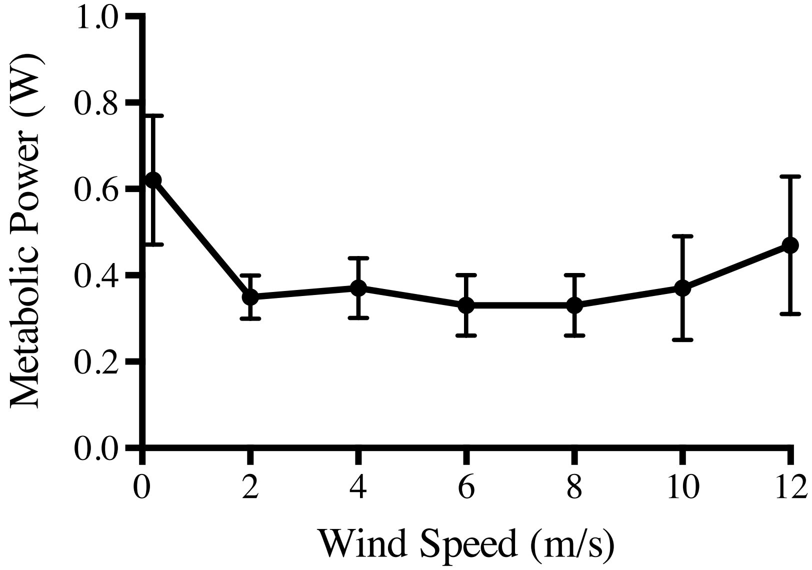

Figure S1. Metabolic power for flight across wind speeds used in this study. The curve exhibits the “U”-shaped pattern seen in other hummingbird species.

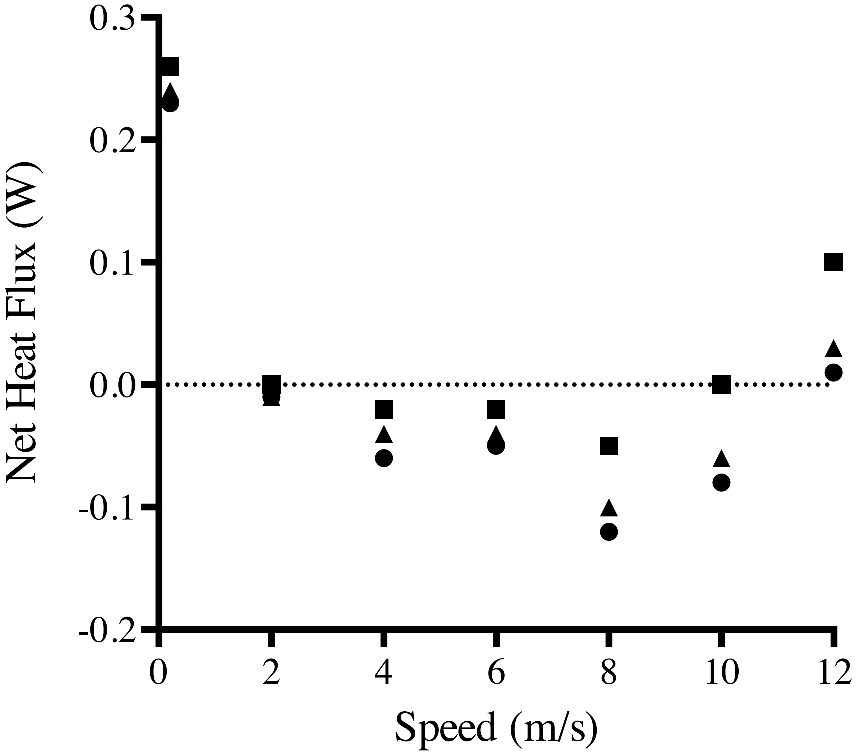

Figure S2. Estimates of net heat flux for *S. calliope* across tested wind speeds assuming heat transfer occurs only across HDAs (~8% of total surface area). Circles are values for H_C (method 1), triangles are values for H_h (method 2), and squares are values for H_S (method 3). The dotted line represents the condition of perfect heat balance
