# Supplementary material for: Heat dissipation during hovering and forward flight in hummingbirds
Source: R Soc Open Sci. 2015 Dec 16;2(12):150598. doi: 10.1098/rsos.150598 (PMC4807464; doi:10.1098/rsos.150598)
Supplement: Table S1 Back Transformed Means [file rsos150598supp2.pdf]

Table S1. Back-transformed predicted means and standard errors from general linear mixed models of mean Ts, maximum Ts and area as a function of flight speed for each heat dissipation region (HDAs).

		Flight Speed (m/s)							Standard Error of Differences		
Measurement	Region	0	2	4	6	8	10	12	Average	Minimum	Maximum
Mean T_s (°C)	Body	24.56	24.06	24.16	24.05	24.00	23.96	24.00	0.3212	0.2330	0.4461
	Hean	30.21	30.17	29.86	29.54	29.85	29.73	29.63	0.1844	0.1318	0.2557
	Feet	29.17	28.05	28.74	28.16	28.48	28.58	28.72	0.6550	0.4865	0.8434
	Axial	30.73	29.66	30.06	29.63	30.14	30.2	30.57	0.2992	0.2323	0.3838
Maximum T_s (°C)	Body	37.65	34.74	34.24	33.91	34.02	34.75	34.76	0.6538	0.6453	0.6751
	Eye	34.92	34.03	33.57	33.22	33.26	33.03	32.67	0.2535	0.2501	0.2619
	Feet	35.28	29.01	28.13	28.01	28.01	28.45	28.01	0.7169	-	-
	Axial	36.28	34.24	33.8	33.9	33.9	35.67	35.55	0.6970	0.6789	0.7422
Area (mm^2)	Eye	45.38	27.97	24.93	23.91	19.01	18.1	14.42	2.559	2.525	2.643
	Axial	54.95	35.39	24.75	21.11	22.1	31.78	22.97	5.617	5.473	5.975
