# Supplementary material for: Heat dissipation during hovering and forward flight in hummingbirds
Source: R Soc Open Sci. 2015 Dec 16;2(12):150598. doi: 10.1098/rsos.150598 (PMC4807464; doi:10.1098/rsos.150598)
Supplement: Table S2 Convection from Sphere Calculations [file rsos150598supp3.pdf]

Table S2. Parameter estimations for calclulation of C_S. Air velocity at 0 m/s was calculated from near-body PIV mid torso.

v (m/s)	*Re*	*Nu*	$h\ (m^2/s)$	T_s
1.52	1342	25.59	43.56	24.95
2	1766	30.17	51.36	24.24
4	3532	45.74	77.84	24.17
6	5297	58.33	99.28	24.05
8	7063	69.32	117.99	23.94
10	8829	79.25	134.89	24.11
12	10595	88.42	150.48	23.65
