# Supplementary material for: Heat dissipation during hovering and forward flight in hummingbirds
Source: R Soc Open Sci. 2015 Dec 16;2(12):150598. doi: 10.1098/rsos.150598 (PMC4807464; doi:10.1098/rsos.150598)
Supplement: Figure S1 Power Curve for Flight [file rsos150598supp4.pdf]

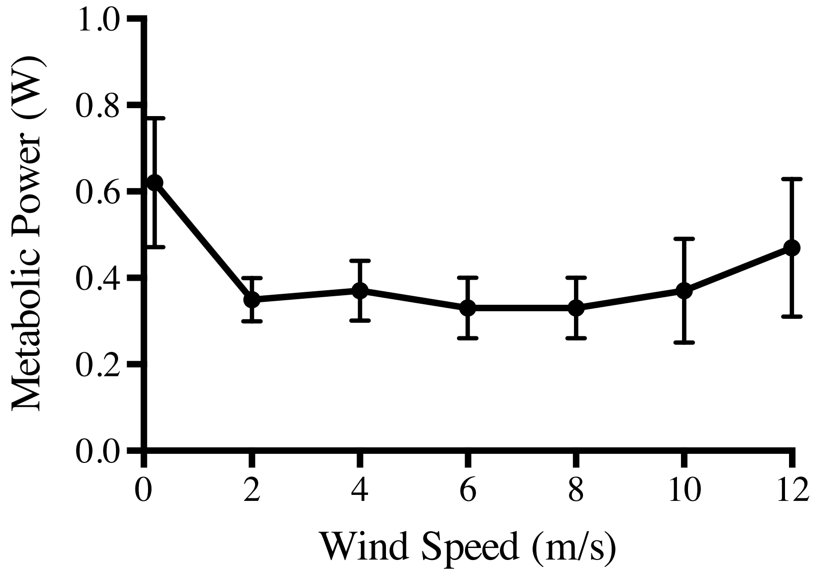

Figure S1. Metabolic power for flight across wind speeds used in this study. The curve exhibits the "U"-shaped pattern seen in other hummingbird species.
